# Mealworm larvae and black soldier fly larvae as novel protein supplements for cattle consuming low-quality forage

**DOI:** 10.1093/tas/txae122

**Published:** 2024-08-13

**Authors:** Mikael N Carrasco, Merritt L Drewery

**Affiliations:** Department of Agricultural Sciences, Texas State University, 601 University Dr., San Marcos, TX 78666, USA; Department of Agricultural Sciences, Texas State University, 601 University Dr., San Marcos, TX 78666, USA

**Keywords:** beef cattle, *Hermetia illucens*, insect protein, protein supplementation, sustainability, *Tenebrio molitor*

## Abstract

The global population is projected to increase, indicating that there will be greater demand for animal protein to meet the associated food needs. This demand will place additional pressure on livestock systems to increase output while also minimizing natural resource inputs. Insect protein has emerged as a potential alternative to conventional protein feeds, such as soybean meal. Mealworm larvae (MWL; *Tenebrio molitor*) have been studied in poultry and swine as an alternative protein source; however, there is no research currently evaluating MWL for cattle. Black soldier fly larvae (BSFL; *Hermetia illucens*) have also received attention for their potential use in livestock feed due to their scalability and nutritional value, but research in cattle is limited. The objective of this study was to evaluate the effects of whole-dried MWL and defatted BSFL as protein supplements for cattle consuming forage. Five ruminally cannulated steers were utilized in a 5 × 5 Latin square experiment to determine how MWL and BSFL supplementations affect forage utilization. Steers consuming ad libitum low-quality forage (76.5% neutral detergent fiber [NDF], 4.2% crude protein) were provided one of the five treatments each period: 1) control with no supplement (CON), 2) soybean meal (CONV), 3) BSFL, 4) MWL, or 5) 50/50 by-weight blend of BSFL and MWL (MIX). All treatments were provided at 100 mg N/kg BW and periods included 8 d for treatment adaptation, 5 d for intake and digestion, and 1 d for ruminal fermentation measures. Protein supplementation stimulated forage organic matter intake (FOMI; *P* ≤ 0.01) relative to CON (3.28 kg/d). There was a significant difference in FOMI (*P* ≤ 0.01) between BSFL (4.30 kg/d) and CONV (4.71 kg/d), but not between CONV and MWL (*P* = 0.06, 4.43 kg/d). Total digestible OM intake (TDOMI) was also stimulated by the provision of protein (*P* ≤ 0.01), from 1.94 kg/d for CON to an average of 3.24 kg/d across protein supplements. Organic matter digestibility (OMD) and NDF digestibility (NDFD) were not affected by treatment (*P* ≥ 0.37), for an average OMD of 66.5% and NDFD of 62.7%. There was also no treatment effect on ruminal volatile fatty acid (*P* = 0.96) or ammonia-N (*P* = 0.22) concentrations. These data indicate that MWL may stimulate forage utilization by beef cattle to a greater extent than BSFL, but both are viable protein supplements.

## Teaser

This research demonstrates that mealworm larvae and black soldier fly larvae can effectively serve as alternative protein sources for cattle consuming forage, enhancing forage intake and digestion to a similar extent as conventional feeds. These findings highlight the potential of insect protein to meet rising global food demands.

## Graphical Abstract

**Graphical Abstract:**
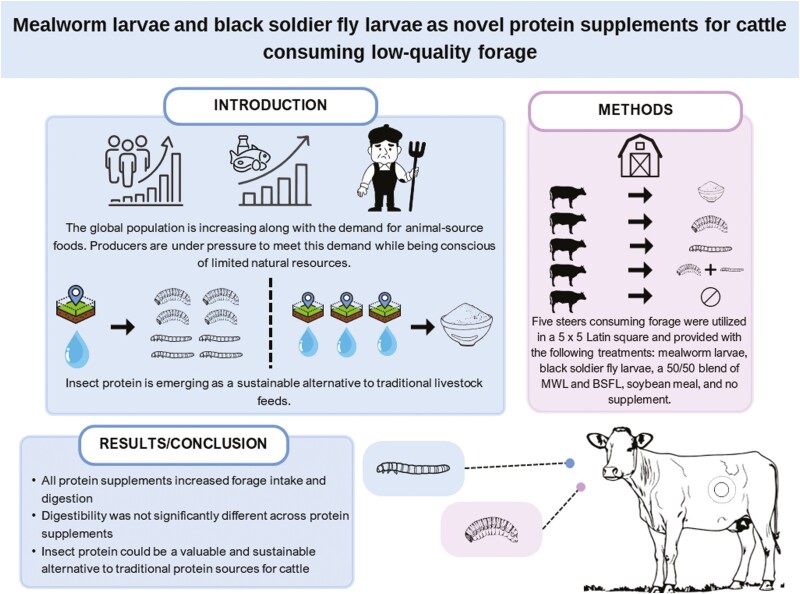

## Introduction

Global food and meat demands are increasing, intensifying the pressure placed on livestock production. To increase production without exacerbating environmental and economic strains, alternatives to conventional livestock feeds should be identified. Insects, such as black soldier fly larvae (BSFL; *Hermetia illucens*) and mealworm larvae (MWL; *Tenebrio molitor*), are receiving attention as alternative livestock feeds due to the efficient use of natural resources and the ability to transform organic wastes into high-quality biomass (van Broekhoven et al., 2015; Zhang et al., 2019; Ruschioni, et al., 2020; Siddiqui et al., 2022). Furthermore, BSFL and MWL have similar nutrient profiles as conventional protein feeds (Spykman et al., 2021) and are commercially produced and approved for some companion animals, fish, swine, and/or poultry in the United States (Hong et al., 2020; Association of American Feed Control Officials [AAFCO], 2022), with ongoing investigations for full integration into the European Union feed supply chain (Liguori et al., 2022).

Although some commercial producers aim to rear and harvest insects as human food, consumers in the United States and other Westernized regions are not likely to accept insects as food (Higa et al., 2020). However, there is consumer support for insects as livestock feed in the United States, especially if there are economic or environmental benefits (Fukuda et al., 2023). Although the economics of utilizing BSFL as a feed in cattle production has been explored (Drewery et al., 2022), large-scale commercial production of edible insects has not yet been realized and it is unclear if insects can be produced at a price point commensurate with conventional feeds.

There have been suggestions that insect production places less pressure on natural resources than conventional food or feed (van Huis and Oonincx, 2017). However, this should be scrutinized and the environmental footprint of insect production should be calculated via life cycle assessments with current rearing practices as insects entering the feed chain in the United States must be reared on feed-grade materials (AAFCO, 2022). While intended for the safe entry of insects into the feed/food chain, this regulation restricts the use of post-consumer food and other organic wastes as rearing substrates, thus limiting the physiological capacity of insects to be reared and harvested as a sustainable feed. Despite this, commercial insect production may create a circular economy by rearing insects on pre-consumer food waste, contributing to waste reduction, and producing protein-rich biomass that can be utilized as feed along with by-products (e.g., frass) that can be placed in other agricultural and related industries (Jasso et al., 2024).

While there has been much inquiry into the viability of edible insects for aquaculture and poultry (Selaledi et al., 2020; Mohan et al., 2022), there is a distinct lack of research on the potential as feed for ruminants. This research is interesting, given the vast scope of cattle production in the United States (U.S. Department of Agriculture [USDA], 2024), the potential of the beef and dairy industries to consume significant amounts of feed, and the unique ability of ruminant animals to upcycle low-quality nutrients and fibrous feed components, such as the chitin found in insect exoskeletons.

For BSFL, there have been in vivo and in vitro trials specifically focused on cattle. In vivo findings indicate that full-fat or defatted BSFL can enhance forage intake without sacrificing digestibility or ruminal fermentation (Fukuda et al., 2022; Tasci et al., 2024). Furthermore, cattle consuming low-quality forage accept BSFL as a stand-alone or blended protein supplement and do not refuse it in amounts that would limit N availability (Tasci et al., 2024). For MWL, ruminal in vitro analyses indicate that inclusion of MWL may decrease diet digestibility (Jayanegara et al., 2017a; Renna et al., 2022; Khanal et al., 2023), but others have reported high ruminal degradability through in situ analyses (Dominguez et al., 2024). To the best of our knowledge, an in vivo feeding trial evaluating MWL in cattle has not been conducted.

Forage quality is essential to maximizing the performance of ruminant animals. Cattle consuming low-quality forage (<7% crude protein, CP) must receive supplemental protein to provide ruminal microbes with adequate N, ultimately increasing fermentation and enhancing forage utilization and concomitant animal performance (Wickersham et al., 2004; Drewery et al., 2014).

The purpose of this project was to add to the repository of literature investigating BSFL as an alternative protein source for cattle and extend this inquiry to another edible insect product, MWL. Accordingly, our objectives were to 1) examine forage utilization responses in steers supplemented isonitrogenous levels of MWL and defatted BSFL and 2) assess whether these novel protein sources stimulate utilization to a similar extent as an isonitrogenous level of a conventional protein source, soybean meal (SBM).

## Materials and Methods

### Data and Sample Collection

These procedures were approved by the Institutional Animal Care and Use Committee at Texas State University (#7726). Five ruminally cannulated steers (291 ± 29 kg) were used in a 5 × 5 Latin square design to analyze intake and digestion in response to MWL and BSFL supplementation to a basal forage diet. During the five experimental periods, one of the five treatments was provided to each steer: 1) SBM, 2) BSFL, 3) MWL, 4) a by-weight 50/50 blend of MWL and BSFL (MIX), and 5) no supplementation (CON). Treatments were calculated to be isonitrogenous and delivered at 100 mg N/kg BW based on previous literature that indicates this dosage maximizes forage utilization responses (Drewery et al., 2014).

Steers were housed in individual pens in a partially enclosed barn and had ad libitum access to fresh water and a trace mineral block. King Ranch Bluestem hay (*Bothriochloa ischaemum*; 4.2% CP; Table 1) was offered daily at 130% of the previous 5-d average consumption.

**Table 1.** Chemical composition of forage, black soldier fly larvae (BSFL), mealworm larvae (MWL), and soybean meal (SBM)

Item	Hay	BSFL	MWL	SBM
	% dry matter			
Organic matter	84.9	90.0	95.8	92.6
Crude protein	4.2	51.5	52.4	49.1
Fat (ether extract)	—	5.3	28.3	—
Neutral detergent fiber	76.5	32.7	19.6	69.0
Acid detergent fiber	54.0	21.6	19.0	15.5
Calcium	0.74	2.07	0.06	0.39
Phosphorus	0.07	1.01	0.72	0.61
Potassium	1.12	1.44	0.84	2.02

Five 14-d experimental periods were conducted: 8 d for treatment adaptation, 5 d for measurement of intake and digestibility, and 1 d for determination of ruminal fermentation parameters. Intake and digestion calculations were made on days 9 through 13. Forage samples were collected on days 9 to 12. Feed refusals (orts) and fecal grab samples were collected every 8 h on days 10 to 13 with sampling time increasing by 2 h every day to obtain samples that were representative of every 2 h within a 24-h period. Acid detergent insoluble ash (ADIA) served as an internal marker to calculate digestibility through estimates of total fecal production on a dry matter (DM) basis.

A rumen fermentation profile was conducted on day 14. Collection of rumen fluid occurred at 0, 4, 8, 12, 16, and 20 h using a suction strainer. Each sample was measured using a portable pH meter. Subsamples of ruminal fluid were prepared and frozen at −20 °C for later determination of volatile fatty acids (VFA) and ammonia-N concentrations. For VFA analysis, 8 mL of rumen fluid was combined with 2 mL of 25% *m*-phosphoric acid, and for ammonia-N analysis, 9 mL of rumen fluid was combined with 1 mL of 1 N HCl.

### Laboratory Analyses

Forage, supplement, feed refusal, and fecal samples were dried at 55 °C in a forced-air oven for 96 h, air-equilibrated, and weighed to determine partial DM. Forage and supplement samples were composited across the day within a period. Feed refusal and fecal samples were composited by steer across the day within a period. Samples were ground with a Wiley Mill to pass a 1-mm screen and then dried at 105 °C for 8 h for DM determination. Organic matter (OM) was subsequently determined as the loss in dry weight upon combustion for 8 h at 450 °C. Nitrogen content was determined with Dumas combustion, and CP was calculated as N × 6.25. Non-sequential neutral detergent fiber (NDF) and acid detergent fiber (ADF) analyses were performed using an Ankom Fiber analyzer with sodium sulfite and amylase omitted and without correction for residual ash. ADIA was determined by combustion of Ankom bags containing ADF residues for 8 h at 450 °C in a muffle furnace.

After thawing, rumen fluid samples were centrifuged at 20,000 × *g* for 20 min and VFA analysis was conducted according to Goestch and Galyean (1983). Specifically, VFA analysis was conducted with an Agilent 7890A gas chromatograph (Agilent Technologies, Santa Clara, CA) equipped with a 30 m × 0.32 mm × 0.25 µm (i.d.) column (Alltech, Deerfield, IL) with an initial column temperature of 80 °C held for 1 min, ramped to 185 °C at 15 °C/min, and then held for 2 min. Injector and detector temperatures were 250 °C. The column flow rate was 2 mL/min, and H_2_ was the carrier gas. For ammonia-N analysis, rumen fluid samples were centrifuged at 3,000 × *g* for 10 min and colorimetric procedures were conducted using UV-vis (Broderick and Kang, 1980).

### Calculations

Fecal production was calculated as kg = [DMI × (ADIA_d_)]/(ADIA_f_) where DMI = DM intake, ADIA_d_ = dietary ADIA concentration (% DM), and ADIA_f_ = fecal ADIA concentration (% DM). Digestibility of DM, OM, and NDF was calculated as Digestibility_x_, (%) = Intake_x_ − Fecal_x_/Intake_x_ × 100 where Intake_x_ = DMI (kg) × dietary concentration of item × (% DM) and Fecal_x_ = Fecal production (kg) × fecal concentration of item × (% DM).

### Statistical Analyses

Intake and digestion were analyzed using the MIXED procedure in SAS 9.4 (SAS Inst. Inc., Cary, NC). Fixed terms in the model were treatment and period with steer as the random effect. Treatment means were calculated with LSMEANS, and the pdiff option was used to separate treatment means. Fermentation profile variables were analyzed using the MIXED procedure. Fixed terms in the model were treatment, period, hour, steer, and treatment × hour and steer included as random terms. The repeated term was hour with treatment × steer as the subject, and the specified covariance structure was compound symmetry based on the Bayesian information criterion. Treatment means were calculated with LSMEANS, and the pdiff option was used to separate treatment means. Statistical significance was determined at *P* ≤ 0.05.

## Results

### Intake and Digestion

There was an effect of treatment on forage OM intake (FOMI; *P* ≤ 0.01; Table 2). Relative to CON, all protein supplements significantly stimulated FOMI (*P* ≤ 0.01). Control steers averaged 3.28 kg FOMI/d, which was less than 4.71, 4.45, 4.43, and 4.30 kg FOMI/d observed for CONV, MIX, MWL, and BSFL, respectively. There was a significant difference in FOMI (*P* ≤ 0.01) between BSFL and CONV, but no other differences between experimental groups.

**Table 2.** Effects of a conventional protein supplement (CONV), black soldier fly larvae (BSFL), mealworm larvae (MWL), or a mixture (MIX) of BSFL and MWL on intake and digestion in cattle consuming forage^*^

	Treatment^†^					SEM	*P*-value
	CON	CONV	BSFL	MWL	MIX		
*n*	5	5	5	5	5		
*OM intake, kg/d*							
Forage	3.28^a^	4.71^b^	4.30^c^	4.43^b,c^	4.45^b,c^	0.16	<0.01
Supplement	0.00^a^	0.32^b^	0.27^c^	0.32^b^	0.29^d^	0.01	<0.01
Total	3.28^a^	5.03^b^	4.57^c^	4.75^b,c^	4.75^b,c^	0.16	<0.01
Digestible	1.94^a^	3.36^b^	3.10^b^	3.19^b^	3.30^b^	0.16	<0.01
*Total tract digestion %*							
DMD	54.3	61.5	62.9	62.1	65.0	3.6	0.30
OMD	60.1	67.0	67.8	67.7	69.7	3.6	0.37
NDFD	57.5	64.3	63.8	62.0	66.1	3.8	0.55

^*^Observations with different subscripts are significantly different at *P* ≤ 0.05.

^†^CON = no supplement; CONV = 100 mg N/kg BW soybean meal; BSFL = 100 mg N/kg BW BSFL; MWL = 100 mg N/kg BW MWL; MIX = 100 mg N/kg BW of 50/50 by-weight blend of MWL and BSFL.

OM, organic matter; DMD, dry matter digestibility; OMD, organic matter digestibility; NDFD, neutral detergent fiber digestibility.

Per experimental design, there was a treatment effect on supplement OM intake (SOMI) relative to CON (*P* ≤ 0.01). Steers receiving CONV, MIX, MWL, or BSFL consumed between 0.27 and 0.32 kg of supplement OM per day, whereas CON steers did not receive supplemental protein. There were significant differences in SOMI between BSFL and either CONV or MWL (*P* ≤ 0.01) and between MIX and either CONV, BSFL, or MWL (*P* ≤ 0.03). The differences observed between protein supplements were a result of the CP content of each and our experimental design to deliver supplements isonitrogenous.

There was a treatment effect on total OM intake (TOMI) between CON and each of the treatments (*P* ≤ 0.01). Steers receiving CON consumed 3.28 kg of total OM per day, which was significantly less than CONV steers (5.03 kg TOMI/d), MIX or MWL steers (4.75 kg TOMI/d), or BSFL steers (4.57 kg TOMI/d). There was a significant difference in TOMI (*P* ≤ 0.01) between CONV and BSFL, but not between other treatment groups.

There was a treatment effect for total digestible OM intake (TDOMI; *P* ≤ 0.01); CONV, MIX, MWL, and BSFL all stimulated TDOMI relative to CON. The CON steers consumed 1.94 kg TDOMI per day, whereas steers receiving CONV, MIX, MWL, and BSFL consumed 3.36, 3.30, 3.19, and 3.10 kg TDOMI per day, respectively. There were no significant differences in TDOMI across steers receiving supplemental protein (*P ≥ *0.27).

Treatment did not affect (*P* = 0.30) DM digestibility (DMD), which averaged 61.1% across all treatments. Organic matter digestibility (OMD) was also not affected by treatment (*P* = 0.37). OMD was 60.1% for CON, 66.9% for CONV, 69.7% for MIX, 67.6% for MWL, and 67.8% for BSFL. Treatment did not affect (*P* = 0.55) NDF digestibility, which was 57.5% for CON steers and ranged from 62.0% to 66.0% for steers receiving supplemental protein.

### Rumen Fermentation Profile

**Figure 1.**
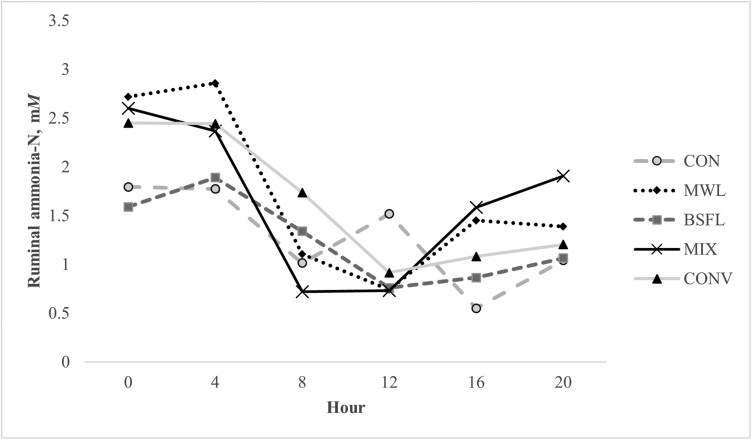
Ruminal ammonia-N concentrations of steers (*n* = 5) fed low-quality forage and supplemented with 0 (CON) or 100 mg N/kg BW of mealworm larvae (MWL), black soldier fly larvae (BSFL), a by-weight 50/50 blend of MWL and BSFL (MIX), or a conventional protein supplement, soybean meal (CONV). Effect of hour (*P* ≤ 0.01). SEM ± 0.35.

Ruminal ammonia concentrations (Table 3) were not affected by treatment × hour (*P* = 0.52), treatment (*P* = 0.70), or period (*P* = 0.10) but were affected by hour (*P* ≤ 0.01; Figure 1) such that ammonia was 2.23 mM at hour 0, increased slightly to 2.27 mM at hour 4, decreased to the lowest concentration at hour 12 (0.93), and then increased gradually through hour 20 (1.31 mM). Across treatment, ruminal ammonia averaged 1.49 mM.

**Table 3.** Ruminal fermentation profile of steers fed low-quality forage supplemented a conventional protein (CONV), black soldier fly larvae (BSFL), mealworm larvae (MWL), or a mixture (MIX) of BSFL and MWL^*^

	Treatment^†^					SEM	*P*-value
	CON	CONV	BSFL	MWL	MIX		
*n*	5	5	5	5	5		
Ammonia-N, mM^‡^	1.01	1.93	1.42	1.58	1.53	0.36	0.70
Total VFA^‖^, mM	108.65	112.22	110.04	110.88	112.34	4.60	0.96
*Molar VFA proportions, %*							
Acetate	75.38	73.94	75.07	74.47	74.28	0.42	0.11
Propionate	15.60	16.04	15.53	16.02	16.11	0.18	0.15
Butyrate	7.51	8.17	7.73	7.65	7.95	0.31	0.37
Isobutyrate	0.59	0.62	0.57	0.62	0.56	0.03	0.28
Valerate	0.42^a^	0.58^b^	0.53^b^	0.60^b^	0.55^b^	0.03	<0.01
Isovalerate	0.47^a^	0.63^b^	0.53^ab^	0.61^bc^	0.52^ac^	0.04	0.03
pH^$^	6.79	6.69	6.76	6.73	6.70	0.02	0.89

^*^Observations with different subscripts are significantly different at *P* ≤ 0.05.

^†^CON = no supplement; CONV = 100 mg N/kg BW soybean meal; BSFL = 100 mg N/kg BW BSFL; MWL = 100 mg N/kg BW MWL; MIX = 100 mg N/kg BW of 50/50 by-weight blend of MWL and BSFL.

^‡^Ruminal ammonia was affected by hour (*P* ≤ 0.01).

^‖^VFA, volatile fatty acid; total VFA was affected by period (*P* ≤ 0.01) and hour (*P* ≤ 0.01).

^$^Rumen pH was affected by period (*P* = 0.04) and hour (*P* ≤ 0.01).

There was no effect of treatment × hour (*P* = 0.15) or treatment (*P* = 0.96) on total VFA, which was 108.65 mM for CON, 112.22 mM for CONV, 112.34 mM for MIX, 110.88 mM for MWL, and 110.04 mM for BSFL. Period (*P* ≤ 0.01) and hour (*P* ≤ 0.01) affected total VFAs. For hour, total VFAs were 115.13 mM at hour 0, decreased to 100.72 mM at hour 4 and 104.46 mM at hour 8, and then returned to 111.14 to 117.36 mM from hours 12 to 20. The significant differences were observed for hour 0 vs. 4 (*P* ≤ 0.01) and hour 0 vs. 8 (*P* = 0.03).

The interaction term, treatment × hour, did not significantly affect the molar proportions of any individual VFA (*P* ≥ 0.18). There was also no effect of treatment on molar proportions of acetate (*P* = 0.11), propionate (*P* = 0.15), butyrate (*P* = 0.37), or isobutyrate (*P* = 0.28), but there was an effect for valerate (*P* ≤ 0.01) and isovalerate (*P* = 0.03).

For valerate, there were significant differences in molar proportions between BSFL vs. CON (*P* = 0.01), CON vs. MIX (*P* ≤ 0.01), CON vs. MWL (*P* ≤ 0.01), and CON vs. SBM (*P* ≤ 0.01). More specifically, valerate comprised 0.43% of VFA for CON, 0.58% for CONV, 0.55% for MIX, 0.60% for MWL, and 0.53% for BSFL. Molar proportions of isovalerate were significantly different between CON vs. MWL (*P* = 0.01), CON vs. CONV (*P* ≤ 0.01), and MIX vs. CONV (*P* = 0.05) such that CON had 0.47% isovalerate, CONV had 0.63%, MWL had 0.62%, and MIX had 0.53%.

Ruminal pH was significantly affected by period (*P* = 0.04) and hour (*P* ≤ 0.01), but not treatment × hour (*P* = 0.82) or treatment (*P* = 0.89). For hour, pH was 6.84 at hour 0, decreased to the lowest observed value at hour 8 (6.64), and then gradually increased to 6.79 through hour 20.

## Discussion

The objectives of this study were to 1) examine forage utilization responses in steers supplemented isonitrogenous levels of MWL and defatted BSFL and 2) assess whether these novel protein sources stimulate utilization to a similar extent as an isonitrogenous level of a conventional protein source, SBM.

Previous research establishes that supplementing protein to low-quality forage (<7% CP) effectively addresses the dietary N deficiency that hinders rumen microbial activity and, thus, the ability of the ruminant animal to utilize forage (Köster et al., 1996; Mathis et al., 2000; Wickersham et al., 2008; Drewery et al., 2021a; Fukuda et al., 2022). In our study, steers consumed forage containing 4.2% CP, thus deeming it low-quality and necessitating additional protein to maximize utilization.

Steers receiving protein supplementation, without regard to the source, consumed more forage OM and total digestible OM as compared to steers receiving only forage, in agreement with previous research (Köster et al., 1996; Wickersham et al., 2008; Drewery et al., 2014, 2021a). As supplements were offered isonitrogenous (100 mg N/kg BW), these findings suggest that MWL and BSFL can enhance forage utilization to a similar extent as a conventional protein supplement, although SBM was associated with the most profound and desired stimulatory effect for FOMI.

Our observation that FOMI was significantly greater for the conventional supplement vs. BSFL contrasts findings of Fukuda et al. (2022), where FOMI was similar for steers consuming low-quality forage and provided with either a full-fat BSFL-based supplement or a conventional protein supplement. Paradoxically, in the current study, we did not observe a significant difference across treatments for TDOMI, while Fukuda et al. (2022) reported that TDOMI was stimulated to a greater extent for the conventional protein supplement than BSFL. The BSFL utilized in the current study was defatted (5.3% ether extract, EE) as compared to the BSFL-based supplement in Fukuda et al. (2022), which was a mixed-ingredient supplement that contained 12.6% EE after blending. Interestingly, steers consuming the insect feed with greater fat content, MWL (28.3% EE), in our study had similar FOMI as those consuming the conventional supplement. Despite this, we do not believe the fat content of insect feeds drives forage utilization responses at levels provided in supplementation scenarios. Furthermore, conventional protein supplements often contain negligible amounts of fat. Rather, perhaps another unique attribute or characteristic of insect feeds impacts forage utilization.

As presented above, supplemental protein increased FOMI; across treatments, the percent increase in FOMI associated with supplementation was 36%. This is greater than that observed by previous researchers who provided the same amount of supplemental protein to low-quality forage as utilized in our study (100 mg N/kg BW) and reported an approximate 14% increase in FOMI relative to steers not receiving supplemental protein (Drewery et al., 2021a; Fukuda et al., 2022). However, our findings align with those of Wickersham et al. (2008) who supplemented rumen degradable protein as casein to low-quality prairie hay. We hypothesize the difference in FOMI response between Fukuda et al. (2022) and Drewery et al. (2021a) vs. our study and Wickersham et al. (2008) is linked to the CP content of the basal forage. The CP of the hay utilized in our trial was 4.2%, which is slightly less than that of Wickersham et al. (2008), 4.9%. Both Fukuda et al. (2022) and Drewery et al. (2021a) fed forage with a slightly greater CP content, approximately 6.6%. As forage CP approaches 7%, additional protein elicits lesser stimulatory responses on FOMI as microbial N requirements are met from the basal diet (Moore and Kunkle, 1995; Köster et al., 1996; Klevesahl et al., 2003). Therefore, the percent increase we observed in FOMI associated with supplementation is consistent with previous research that utilized a basal forage with a similar CP content as ours.

In our study, digestibility was not impacted by supplementation, which agrees with previous in vivo research by Tasci et al. (2024) where an isonitrogenous comparison of defatted BSFL and a conventional protein supplemented to low-quality forage did not reveal differences in DMD, OMD, or neutral detergent fiber digestibility between treatments or as compared to no supplementation. In research with defatted algae, Drewery et al. (2021a) also did not observe a change in OMD associated with protein supplementation. The lack of a treatment effect on digestion was expected in the present study due to the competing effects of passage and digestion. Protein supplementation increases low-quality forage intake which, accordingly, increases passage rate (McCollum and Horn, 1990; Köster et al.,1996; Mathis et al., 2000). When digesta is retained in the rumen for a shorter period, there is less potential for microbes to interact with dietary OM. However, when low-quality forage is fed without additional protein, there is less fermentative activity due to microbial N requirements not meeting addressed. Therefore, the effects of increased intake and passage rate associated with protein supplementation likely canceled out the effects of increased microbial activity in our study, resulting in a net similar impact on digestibility across treatments.

Jayanegara et al. (2017b) incubated BSFL with grasses and forages in an in vitro ruminal fermentation system and observed a depression in in vitro DMD and OMD which they attributed to the chitin content of BSFL. In a separate study, Jayanegara et al. (2017a) reported lower in vitro OMD for MWL and BSFL vs. SBM, which was also attributed to the chitin content of the insect feeds. There are limitations to in vitro studies, and true digestibility is often underestimated with these analyses (Nolan et al., 2023). Therefore, in vitro systems often cannot fully mimic the complexity of intake and digestion, especially when these physiological processes are driven by rumen microbial populations that respond to dietary shifts and other environmental conditions. It is likely that the methodological approach and associated analyses explain the differences in our findings for in vivo digestibility vs. those in other studies that utilize in vitro systems.

Insects are unique from conventional livestock feeds in that they contain chitin, an acetylated polysaccharide with a similar molecular structure as cellulose, in their exoskeletons and exuviae. Chitin is generally present in the greatest and consistent amounts across the larval, prepupae, and pupae phases, before the insect reaches adulthood (Soetemans et al., 2020). These are the life stages during which edible insects, including BSFL and MWL, are often harvested. Estimates of nutrient content, including chitin, in different insect species vary and are impacted by rearing conditions, such as diet and environmental temperature (Adámková et al., 2017). The chitin content of BSFL is estimated to be 6% to 8% (Zlotko et al., 2021), whereas that of MWL is 5% to 13% (Adámková et al., 2017; Song et al., 2018).

Previous research has posited that the chitin content of insects may hinder digestibility when fed to livestock; however, the current repository of literature on insects as feed focuses on monogastric livestock and poultry. Therefore, the perceived challenge of chitin as a hindrance to digestibility may be overstated and/or irrelevant for ruminant animals, which do not solely utilize auto-enzymatic digestion in the true stomach and small intestine but rely mainly on rumen microorganisms that can be fibrolytic and chitinolytic (Kopecny et al., 1996). There is research about chitosan, the partially or fully deacetylated version of chitin, in ruminant diets, but the literature on the effects of dietary chitin on intake, digestion, and fermentation is scant (Shah et al., 2022). While we did not analyze our supplements for chitin, digestibility was not different across treatment groups, indicating that the chitin naturally present in BSFL and MWL does not negatively affect digestibility when these insects are fed as protein supplements. However, there is likely an upper limit of inclusion beyond which the chitin component of insects would hinder digestibility—while this threshold would likely not be met for cattle receiving insect feeds as protein supplements, it may be a concern in feedlot and/or dairy rations for which BSFL or MWL would reasonably be consumed at greater quantities than in our study. Future research could investigate replacing protein sources and concentrates in total mixed rations with BSFL or MWL and establish inclusion rates that do not sacrifice animal performance.

Ruminal fermentation parameters, including total VFA, molar proportions of major VFA, ruminal ammonia, and rumen pH, were comparable to previous research that has implemented similar experimental diets and treatments (Fukuda et al., 2022; Tasci et al., 2024). This further indicates that BSFL and MWL do not disrupt normal rumen physiology and fermentation when provided to cattle consuming forage.

Ultimately, data from this trial add to the emerging literature on the viability of insect feeds in ruminant production systems. Our research is the first in vivo trial evaluating the suitability of MWL as a protein supplement for cattle, opening an additional line of inquiry for a novel insect product to be incorporated into the cattle feeding industry. This study is limited by the confounding effect of the fat content of the protein supplements; while supplements were provided isonitrogenous, they were not isoenergetic. However, we do not believe fat content was a significant driver of our findings given the relatively low level of supplementation. As the nutritional value of insects depends on the rearing substrate and other processing parameters, future research should link upstream processing to the value and quality of downstream products (Drewery et al., 2021b) with the overarching goal of identifying optimal processing procedures that maximize nutrient digestibility for livestock.
